# Worst-Case Cooperative Jamming for Secure Communications in CIoT Networks

**DOI:** 10.3390/s16030339

**Published:** 2016-03-07

**Authors:** Zhen Li, Tao Jing, Liran Ma, Yan Huo, Jin Qian

**Affiliations:** 1 School of Electronic and Information Engineering, Beijing Jiaotong University, Beijing 10044, China; 12111041@bjtu.edu.cn (Z.L.); yhuo@bjtu.edu.cn (Y.H.); 12111020@bjtu.edu.cn (J.Q.); 2 Department of Computer Science, Texas Christian University, Fort Worth, TX 76129, USA; l.ma@tcu.edu

**Keywords:** CIoT networks, physical layer security, cooperative jamming, incentive mechanism, Vickrey auction

## Abstract

The Internet of Things (IoT) is a significant branch of the ongoing advances in the Internet and mobile communications. Yet, the use of a large number of IoT devices can severely worsen the spectrum scarcity problem. The usable spectrum resources are almost entirely occupied, and thus, the increasing demands of radio access from IoT devices cannot be met. To tackle this problem, the Cognitive Internet of Things (CIoT) has been proposed. In a CIoT network, secondary users, *i.e.*, sensors and actuators, can access the licensed spectrum bands provided by licensed primary users (such as cellular telephones). Security is a major concern in CIoT networks. However, the traditional encryption method at upper layers (such as symmetric and asymmetric ciphers) may not be suitable for CIoT networks since these networks are composed of low-profile devices. In this paper, we address the security issues in spectrum-leasing-based CIoT networks using physical layer methods. Considering that the CIoT networks are cooperative in nature, we propose to employ cooperative jamming to achieve secure transmission. In our proposed cooperative jamming scheme, a certain secondary user is employed as the helper to harvest energy transmitted by the source and then uses the harvested energy to generate an artificial noise that jams the eavesdropper without interfering with the legitimate receivers. The goal is to minimize the Signal to Interference plus Noise Ratio (SINR) at the eavesdropper subject to the Quality of Service (QoS) constraints of the primary traffic and the secondary traffic. We formulate the minimization problem into a two-stage robust optimization problem based on the worst-case Channel State Information of the Eavesdropper (ECSI). By using Semi-Definite Programming (SDP), the optimal solutions of the transmit covariance matrices can be obtained. Moreover, in order to build an incentive mechanism for the secondary users, we propose an auction framework based on the cooperative jamming scheme. The proposed auction framework jointly formulates the helper selection and the corresponding energy allocation problems under the constraint of the eavesdropper's SINR. By adopting the Vickrey auction, truthfulness and individual rationality can be achieved. Simulation results demonstrate the effective performance of the cooperative jamming scheme and the auction framework.

## 1. Introduction

With the tremendous deployment of the Internet and mobile communications, the Internet of Things (IoT) emerges as a promising technology to connect billions of everyday objects and the surrounding environments using different kinds of sensors and actuators [1,2,3]. The use of a large number of IoT devices leads to a far more serious problem in terms of spectrum scarcity [4]. To solve this, a new paradigm termed the Cognitive Internet of Things (CIoT), which employs the cognitive radio technology in IoT networks, has been advocated [5,6,7]. Within a CIoT network, an unlicensed secondary user (an IoT device, such as a sensor or a actuator) is capable of operating on the licensed spectrum bands provided by a licensed primary user (such as a telephone). There are generally two types of CIoT networks: (1) spectrum-sensing-based CIoT networks [8] and (2) spectrum-leasing-based CIoT networks [9]. In the spectrum-sensing-based CIoT networks, spectrum sensing is an indispensable part. Yet, spectrum sensing may be inaccurate and leads to high power consumption [10]. Instead of employing spectrum sensing, in the spectrum-leasing-based CIoT networks, a secondary user may cooperatively assist a primary user to relay the primary signals; and in return, the primary user leases its spectrum and reserves a period of time for the secondary user to transmit its data. Moreover, if the secondary user is equipped with a multi-antenna, it can relay the primary signals while concurrently transmitting its own data.

Just like traditional wireless networks, security is one of the fundamental concerns in CIoT networks [11] (unless otherwise specified, the CIoT networks mentioned below refer to spectrum-leasing-based CIoT networks). The primary user has the requirement to prevent the eavesdropping attack (there are many different attack modes, such as denial-of-service (DoS) attacks, replay attacks and eavesdropping attacks; in this paper, we pay attention to the eavesdropping attack). In order to achieve secure transmission, physical (PHY) layer security, which exploits the characteristics of the radio spectrum to limit the amount of information extracted by eavesdroppers, has been proposed [12,13,14,15,16,17,18]. Compared to the traditional encryption methods in the upper layer, physical layer security has its advantages [19,20]. In CIoT networks with low-complexity devices, some issues are raised in upper layer security, such as key distribution for symmetric cryptosystems and the high computational complexity of asymmetric cryptosystems [21]. Different from upper layer security, physical layer security schemes enable secret communication without the aid of an encryption key. Meanwhile, the computational complexity of physical layer security schemes is low [19]. Moreover, all encryption methods are based on the assumption that it is computationally infeasible for the eavesdropper since the computing capacity of the eavesdropper is limited [22]. However, this assumption is not suitable due to the growth of computational power. For physical layer security, no limitations are assumed for the eavesdropper in terms of computational resources. For the above reasons, physical layer security is more suitable for CIoT networks. Considering that CIoT networks are cooperative networks, it is a logical idea to employ cooperation techniques in physical layer security to improve the security capability. One of the cooperation techniques is cooperative jamming, and it has been widely studied in other networks [16,17,18]. The main idea of cooperative jamming is to generate an artificial noise via multiple antennas on a cooperative helper (such as a secondary user) to confuse the eavesdropper.

However, these cooperative jamming schemes cannot be directly used in CIoT networks, since an incentive mechanism is lacking for the secondary user, which acts as the cooperative helper. As we all know, the secondary users and the primary users generally belong to different authorities [4]. For example, in the 802.22 standard [23], the secondary users that belong to the mobile phone operator can use the TV bands that are owned by the TV operator, if they cause no interference with the pay-TV subscribers (the primary users). Thus, it is unrealistic to assume that they would cooperate with each other unconditionally. To be more specific, as mentioned before, in CIoT networks, although a certain secondary user spends its own power to relay the primary signals, the secondary user gets the right to use the spectrum resource in return (the incentive mechanism of the relay node is out of the scope of this paper [27]). Additionally, for the secondary user, who acts as the helper, it uses its own power resource to jam the eavesdropper, while getting no return. On the other hand, a preferable strategy for a rational primary user is to pay less reward to the helper, while keeping a desired security capability. The problem of how to choose and negotiate with a suitable helper among multiple secondary users needs to be solved.

Bearing these challenges in mind, at first, we propose a novel cooperative jamming scheme to encourage the helper by employing energy harvesting technology [24]. In our scheme, the transmission is separated into two phases. In the first phase, a certain secondary user is arbitrarily assigned as the helper and harvests energy provided by a primary transmitter (here, we assume all of the secondary users (the IoT devices) have the energy transfer capability). In the second phase, the helper sends jamming signals to jam the eavesdropper using the energy harvested in the first phase. With the assistance of multiple antennas, the helper adjusts its transmit covariance matrix to jam the eavesdropper and simultaneously nulls out the interference at legitimate receivers. The transmit covariance matrix is an important parameter in multi-antenna-based networks. Suppose the signal vector sent by the helper is x. Then, the transmit covariance matrix Q can be formulated as Q = $E[xx^{†}]$. The goal of the proposed scheme is to deteriorate the signal to interference plus noise ratio (SINR) at the eavesdropper with quality of service (QoS) constraints on the SINR of the primary traffic and the secondary traffic. It is worth mentioning that most of the research in this area assumes that the transmitters can get the perfect Channel State Information of the Eavesdropper (referred to as ECSI) [25,26]. However, this assumption appears to be too ideal, because the eavesdropper, despite being a legitimate user, wishes to hide from the transmitter without being cooperative in the stage of channel estimation. Thus, only imperfect ECSI can be known. In this paper, we assume there exists channel mismatches for the eavesdropper links that are norm-bounded [28,29].

Based on this scheme, we propose a novel auction framework to solve the helper selection and the corresponding energy allocation problems. In the proposed auction framework, the primary user acts as the auctioneer, and the secondary users act as the bidders. The winning bidder is employed as the helper. Instead of bidding by virtual money, a nonmonetary bidding language is designed to let the users participate by a barter-like exchange. To be more specific, the primary user provides the power resource to the helper in exchange for a deteriorated SINR at the eavesdropper, and the secondary users compete for the power resource by cooperatively jamming the eavesdropper. The proposed framework guarantees an improved secure communication quality of the primary user, *i.e.*, the degraded SINR at the eavesdropper, and stimulates the cooperative behavior of the helper. Finally, we evaluate the performance of our proposed jamming scheme and the auction framework via comprehensive simulation studies.

The rest of the paper is organized as follows. Section 2 discusses the related work. Section 3 presents the CIoT network model and describes its communication processes. The cooperative jamming scheme is detailed in Section 4. Section 5 describes the design of the auction framework. Section 6 evaluates the performance of the jamming scheme and the auction framework. Section 7 concludes the paper.

Notations: In this paper, the bold capital and lower-case letters are used to denote matrices and vectors, respectively. ${\left(\cdot \right)}^{†}$, Tr(·), and ||·|| denote the Hermitian transpose of a matrix, trace and the Euclidean norm, respectively. E[·] is the statistical expectation. I denotes an identity matrix of corresponding dimension. $X\in S^{N}$ and $Y\in S_{+}^{N}$ represent X as an n-dimension symmetric matrix and Y as an *n*-dimension positive semi-definite matrix, respectively.

## 2. Related Work

In this section, we summarize the related work under the categories of jamming schemes in physical layer security and auction frameworks in resource allocation.

### 2.1. Existing Work on Jamming in Physical Layer Security

Jamming is a common method to protect security in wireless networks. The authors in [12] first attempted to use artificial noise to degrade the eavesdropper’s channel, while this does not affect the channel of the intended receiver. Following this work, considerable research has investigated jamming with multiple antennas [13,14,15]. In these schemes, in addition to the information signal, part of the transmit power was allocated to send jamming signals. However, these approaches may be less effective in cooperative networks, since the cooperative characteristic in these networks is not effectively used. To enhance the security performance in cooperative networks, friendly helpers are employed to send jamming signals to confuse the eavesdropper [16,17,18]. This approach is often referred to as cooperative jamming. Cooperative jamming was initiated in [16]. The authors proposed a cooperative jamming scheme in the general Gaussian multiple access wire-tap channel and the Gaussian two-way wire-tap channel, where a non-transmitting user can help increase the secrecy capacity for a transmitting user by jamming the eavesdropper. Huang *et al.* [17] solved the physical layer security issue in relay networks by using cooperative jamming. To be more specific, the normally inactive node in the relay network was employed as the cooperative jamming source to confuse the eavesdropper. The closed-form jamming beamformers and the corresponding optimal power allocation of the employed node were derived in this work. Araujo *et al.* [18] introduced cooperative jamming in cognitive wireless sensor networks. Three scenarios were considered in this work: eavesdropper location known, eavesdropper location unknown and eavesdropper and relay co-location. The approach applied was similar to cooperative jamming [16].

Inspired by [16,17,18], we attempt to introduce cooperative jamming in CIoT networks. Note that these schemes are applied in the traditional wireless networks, and energy consumption is not considered as a design constraint. Nevertheless, for CIoT networks, energy consumption is of the utmost importance, since the CIoT devices are low-energy [30]. It is unrealistic to assume that the cooperative nodes would cooperate with each other unconditionally due to the limited energy resource. This literature cannot be directly used in CIoT networks, since an incentive mechanism is lacking to stimulate the CIoT devices. Zhang *et al.* [31] proposed an incentive mechanism in spectrum-leasing-based cognitive radio networks, whereby the primary user cooperates with two individual secondary users. In the scheme, these two secondary users act as a relay and a cooperative helper to improve the primary user’s secrecy. In return, the primary user allocates a fraction of access time for the secondary users’ transmission. Moreover, the case where the primary user cooperates with a cluster of secondary users to enhance the secrecy via collaborative beamforming was also considered. This inspiring work used the right of channel access as the reward. However, the helper node may not need to use the channel at the particular time slot. In addition, a simple case where each node is only equipped with one antenna was considered.

As mentioned before, in CIoT networks, the energy consumption is an important issue [30]. To solve it, energy harvesting technology has been introduced in CIoT networks [32,33,34]. Inspired by this, using energy as the reward for the helper in CIoT networks becomes a reality and may be a possible solution for the incentive mechanism. Xing *et al.* [35] first proposed a harvest-and-jam scheme, via which multi-antenna helpers harvest energy in the first transmission phase and jam the eavesdropper by consuming the harvested energy in the second transmission phase. In this literature, the helper candidates are deployed sufficiently close to the transmitter to reduce the power cost due to the relentless path loss. Moreover, the helper is randomly selected and uses all of the harvested energy to jam the eavesdropper. Thus, how to jointly select the cooperative helper and allocate the corresponding energy resource are still a whitespace in CIoT networks.

### 2.2. Existing Work on Auction

As we know, auction has been widely applied to resource allocation in wireless networks. Li *et al.* [36] proposed a spectrum-management framework based on auction theory to achieve a win-win situation for primary users and secondary users. Zou *et al.* [37] studied a power allocation scheme based on auction theory, in which the transmitting power is considered as the trading good. Wang *et al.* [38] considered the resource allocation problem for operational grids and networks under the constraint of a commercially-offered resource. With the purpose of avoiding misuse potentials, a trust-incentive-based combinatorial double auction algorithm was developed. The core idea of this paper is to adopt each peer’s trust values to adjust their bids. To challenge the monopoly of mobile termination charges, Tsiaras *et al.* [39] proposed an auction-based charging and user-centric system. The Vickrey auction was applied to guarantee honest bids that participants are forced to make. However, these research works assumes the use of monetary gains, *i.e.*, virtual currency or credit to stimulate the participators in an auction. Thus, the method leads to problems in terms of money transaction and more complicated implementation. To solve this, the barter-like resource exchange-based auction framework has been put forward [40]. Of particular relevance to our work is [40], which addressed the physical layer security issue in spectrum-leasing-based cognitive radio networks by employing auction theory. The authors proposed a nonmonetary trading model, where the primary user creates the transmission opportunity for the secondary user, and the secondary user can choose to act as a relay or a helper to improve the secrecy rate of the primary user. Different from this inspiring work, our paper employs energy harvesting technology and uses energy as the payment to stimulate the helper.

## 3. System and Communication Models

**Figure 1:**
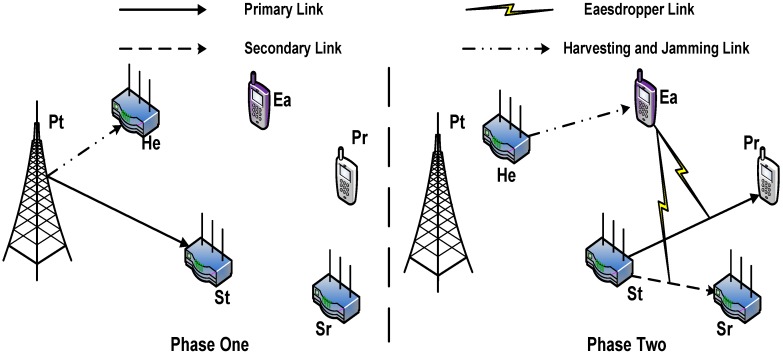
System model.

As shown in Figure 1, our CIoT network is made up of two subnetworks. The primary network (a cellular network) consists of a primary transmitter-receiver pair (Pt, Pr), while the secondary network (an IoT network) consists of a secondary transmitter-receiver pair (St, Sr) and a secondary user that acts as a helper (He) (note that there exits a large number of CIoT devices in the secondary network; here, we only introduce three of them for clarity). Pt intends to communicate with Pr in the the presence of an eavesdropper (Ea). Without loss of generality, we assume to employ St to relay the primary traffic. St, Sr and He are equipped with multiple antennas, while Pt, Pr and Ea are legacy devices with a single antenna. The number of antennas of St, Sr and He are denoted by $N_{St}$, $N_{Sr}$, and $N_{He}$, respectively. Since we assume each node is half-duplex, the transmission in our model is separated into two phases. In the first phase, Pt sends its information signals to St while simultaneously transferring energy to He. In the second phase, St simultaneously forwards the information signals to Pr and transmits its own signals to Sr, respectively. Meanwhile, He performs cooperative jamming using the respective harvested energy from the first transmission phase. Due to the distance constraint, similar to [42,43], we focus on the scenario that a direct transmission link cannot be set up between Pt and Pr, between Pt and Sr and between Pt and He. Note that in our network model, in a certain time slot, the channel provided by the primary user can only be occupied by one secondary transmitter-receiver pair. Thus, the interference caused by the helper cannot impact other devices. Similar to [44], in the following subsections, we expound the detailed procedure of these two phases.

### 3.1. Communication Process in Phase 1

In the first transmission phase, Pt sends its information signals to St while simultaneously transferring energy to He. The signals received at He and St can be expressed as:

(1) $$y_{H_{e}}=h_{P_{t}H_{e}}s_{P_{t}}+n_{H_{e}}$$

(2) $$y_{S_{t}}=h_{P_{t}S_{t}}s_{P_{t}}+n_{S_{t}}$$

where $s_{P_{t}}$ denotes the information signals sent by Pt. We express the power allocation of Pt as $E[|s_{P_{t}}{|}^{2}]=P_{P_{t}}$, where $P_{P_{t}}$ is the transmit power constraint on Pt. $n_{H_{e}}\in \mathrm{CN}\left(0,N_{A}I\right)$ and $n_{S_{t}}\in \mathrm{CN}\left(0,N_{A}I\right)$ are modeled as zero mean additive white Gaussian noise (AWGN) with variances of $N_{A}I$; $h_{P_{t}H_{e}}$ and $h_{P_{t}S_{t}}$ represent the channel coefficient vectors from Pt to He and from Pt to St, respectively.

On the other hand, for the wireless power transfer, the harvested energy of He is given by:

(3) $$E_{H_{e}}=\eta E[∥h_{P_{t}H_{e}}s_{P_{t}}{∥}^{2}]=\eta P_{P_{t}}{∥h_{P_{t}H_{e}}∥}^{2}$$

where 0≤η<1 denotes the energy harvesting efficiency of He.

### 3.2. Communication Process in Phase 2

We assume St employs the decode-and-forward (DF) protocol due to its simplicity of presentation. In the second transmission phase, St simultaneously forwards the information signals to Pr and transmits its own signals to Sr. In the same time, He transmits jamming signals to jam Ea. The signals received at Pr, Sr and Ea can be written as:

(4) $$y_{P_{r}}=h_{S_{t}P_{r}}s_{P_{t}}+h_{S_{t}P_{r}}s_{S_{t}}+h_{H_{e}P_{r}}s_{H_{e}}+n_{P_{r}}$$

(5) $$y_{S_{r}}=H_{S_{t}S_{r}}s_{P_{t}}+H_{S_{t}S_{r}}s_{S_{t}}+H_{H_{e}S_{r}}s_{H_{e}}+n_{S_{r}}$$

(6) $$y_{E_{a}}=h_{S_{t}E_{a}}s_{P_{t}}+h_{S_{t}E_{a}}s_{S_{t}}+h_{H_{e}E_{a}}s_{H_{e}}+n_{E_{a}}$$

Without ambiguity, the primary signals are still denoted by $s_{P_{t}}$. $s_{S_{t}}$ and $s_{H_{e}}$ represent the secondary signals sent by St and the jamming signals sent by He, respectively. The transmit covariance matrices of St and He are written as $Q_{S_{t}1}$=$E[|s_{P_{t}}{|}^{2}]$, $Q_{S_{t}2}$=$E[|s_{S_{t}}{|}^{2}]$ and $Q_{H_{e}}$=$E[|s_{H_{e}}{|}^{2}]$, respectively. The power constraints are denoted by $\mathrm{Tr}\left(Q_{S_{t}1}\right)\leq P_{S_{t1}}$, $\mathrm{Tr}\left(Q_{S_{t}2}\right)\leq P_{S_{t2}}$ and $\mathrm{Tr}\left(Q_{H_{e}}\right)\leq P_{H_{e}}$, respectively. $n_{P_{r}}$, $n_{S_{r}}$ and $n_{H_{e}}$ represent the noises at Pr, Sr and Ea, respectively; and $H_{ij}$ ($h_{ij}$) refers to the channel coefficient matrix/vector from node *i* to node *j*, where i∈{St,He} and j∈{Pr,Sr,Ea}. As previously mentioned, in this paper, we consider the case when only imperfect ECSI can be known. We propose to use a norm-bounded model to characterize the uncertainties of ECSI, such that:

(7) $$H_{S_{t}E_{a}}=\left\{h_{S_{t}E_{a}}|h_{S_{t}E_{a}}={\hat{h}}_{S_{t}E_{a}}+\Delta h_{S_{t}E_{a}}\right\}$$

(8) $$H_{H_{e}E_{a}}=\left\{h_{H_{e}E_{a}}|h_{H_{e}E_{a}}={\hat{h}}_{H_{e}E_{a}}+\Delta h_{H_{e}E_{a}}\right\}$$

where ${\hat{h}}_{S_{t}E_{a}}$ and ${\hat{h}}_{H_{e}E_{a}}$ are the estimates of the corresponding channels; $\Delta h_{S_{t}E_{a}}$ and $\Delta h_{H_{e}E_{a}}$ are their respective channel errors. Here, we assume that the channel errors consist of the norm-bounded sets $E_{1}=\{\Delta h_{S_{t}E_{a}}:∥\Delta h_{S_{t}E_{a}}{∥}^{2}\leq \epsilon_{1}^{2}\}$ and $E_{2}=\{\Delta h_{H_{e}E_{a}}:∥\Delta h_{H_{e}E_{a}}{∥}^{2}\leq \epsilon_{2}^{2}\}$, where $\epsilon_{1}^{2}$ and $\epsilon_{2}^{2}$ are known constants.

To remove the interference caused by jamming signals, as well as primary signals, since Sr is equipped with multiple antennas, we can deliberately design a decoding vector $v_{S_{r}}$ to decode the secondary signals at Sr, by letting:

(9) $$v_{S_{r}}^{†}H_{S_{t}S_{r}}s_{P_{t}}=0$$

(10) $$v_{S_{r}}^{†}H_{H_{e}S_{r}}s_{H_{e}}=0$$

Therefore, the secondary signals received at Sr can be formulated as:

(11) $$y_{S_{r}}=v_{S_{r}}^{†}H_{S_{t}S_{r}}s_{S_{t}}+v_{S_{r}}^{†}n_{S_{r}}$$

As for the signals received at Pr, since Pr has only one antenna, it does not have the capability to apply the decoding vector. To remove the undesirable interference at Pr, we propose to use zero-forcing (ZF) (which has been widely used in multi-antenna-based networks [45]) at St and He, since these two transmitters are equipped with multiple antennas. We can deliberately design the transmit covariance matrices by setting $h_{S_{t}P_{r}}Q_{S_{t}2}h_{S_{t}P_{r}}^{†}=0$ and $h_{H_{e}P_{r}}Q_{H_{e}}h_{H_{e}P_{r}}^{†}=0$. Then, the received primary signals at Pr can be written as:

(12) $$y_{P_{r}}=h_{S_{t}P_{r}}s_{P_{t}}+n_{P_{r}}$$

A detailed derivation of $Q_{S_{t}1}$, $Q_{S_{t}2}$ and $Q_{H_{e}}$ will be given in the next section. In summary, we meticulously describe the two communication processes in our CIoT network model in this section. One can see that the undesirable interference caused by $H_{e}$ is successfully removed. Furthermore, both the primary signals and the secondary signals can be perfectly received at Pr and Sr, respectively.

## 4. Proposed Cooperative Jamming Scheme

In this section, we aim to deteriorate the SINR at Ea with QoS constraints of the primary traffic and the secondary traffic by designing the transmit covariance matrices, *i.e.*, $Q_{S_{t}1}$, $Q_{S_{t}2}$ and $Q_{H_{e}}$ (note that, in the scheme, we assume He uses all of the harvested energy to jam Ea; helper selection and the corresponding energy allocation problems will be solved in the next section). We formulate this problem into a two-stage robust optimization problem and use semi-definite programming (SDP) methods to solve it.

### 4.1. Problem Formulation

The SINR at Ea can be written as:

(13) $$\gamma_{E_{a}}=\frac{\left({\hat{h}}_{S_{t}E_{a}}+\Delta h_{S_{t}E_{a}}\right)\left(Q_{S_{t}2}+Q_{S_{t}1}\right){\left({\hat{h}}_{S_{t}E_{a}}+\Delta h_{S_{t}E_{a}}\right)}^{†}}{\left({\hat{h}}_{H_{e}E_{a}}+\Delta h_{H_{e}E_{a}}\right)Q_{H_{e}}{\left({\hat{h}}_{H_{e}E_{a}}+\Delta h_{H_{e}E_{a}}\right)}^{†}+N_{A}}$$

The constraints on the SINR of Pr and Sr can be simply presented as:

(14) $$\frac{h_{S_{t}P_{r}}Q_{S_{t}1}h_{S_{t}P_{r}}^{†}}{N_{A}}\geq \gamma_{P_{r}}$$

(15) $$\frac{\mathrm{Tr}(H_{S_{t}S_{r}}Q_{S_{t}2}H_{S_{t}S_{r}}^{†})}{N_{A}}\geq \gamma_{S_{r}}$$

where $\gamma_{P_{r}}$ and $\gamma_{S_{r}}$ represent the SINR of Pr and Sr, respectively.

Now, we focus on obtaining robust transmit covariance matrices based on the worst-case ECSI. Additionally, the problem of minimizing the SINR at Ea can be formulated as:

(16) $$\begin{array}{cc} \min_{Q_{S_{t}1},Q_{S_{t}2},Q_{H_{e}}}\;\max_{\Delta h_{S_{t}E_{a}},\Delta h_{H_{e}E_{a}}} & \gamma_{E_{a}} \end{array}$$

(17) $$\begin{array}{cc} s.t.\; & \frac{h_{S_{t}P_{r}}Q_{S_{t}1}h_{S_{t}P_{r}}^{†}}{N_{A}}\geq \gamma_{P_{r}} \end{array}$$

(18) $$\begin{array}{cc} & \frac{\mathrm{Tr}(H_{S_{t}S_{r}}Q_{S_{t}2}H_{S_{t}S_{r}}^{†})}{N_{A}}\geq \gamma_{S_{r}} \end{array}$$

(19) $$\begin{array}{cc} & h_{S_{t}P_{r}}Q_{S_{t}2}h_{S_{t}P_{r}}^{†}=0 \end{array}$$

(20) $$\begin{array}{cc} & h_{H_{e}P_{r}}Q_{H_{e}}h_{H_{e}P_{r}}^{†}=0 \end{array}$$

(21) $$\begin{array}{cc} & \eta P_{P_{t}}{∥h_{P_{t}S_{t}}∥}^{2}\geq \mathrm{Tr}\left(Q_{H_{e}}\right) \end{array}$$

where Equations (19) and (20) imply the ZF constraints to null out the secondary signals and the jamming signals at Pr, respectively, and Equation (21) indicates that He consumes all of the energy harvested in the first phase to jam Ea in the second phase. We will study how to solve this problem in the next subsection.

### 4.2. Proposed Solutions

This optimization problem consists of a linear fractional objective function and a set of affine inequalities and equalities. Thus, it is a quasi-convex problem, which is hard to solve. In this paper, we relax some constraints and reformulate this problem into a two-stage optimization problem.

#### 4.2.1. The First Stage

In the first stage, first, we make a relaxation of Equation (18) by setting:

(22) $$\frac{\mathrm{Tr}(H_{S_{t}S_{r}}Q_{S_{t}2}H_{S_{t}S_{r}}^{†})}{N_{A}}=\gamma_{S_{r}}$$

Then, we aim to minimize the part that is related to $Q_{S_{t}2}$ in Equation (13). Additionally, we have:

(23) $$\begin{array}{cc} \min_{Q_{S_{t}2}}\max_{\Delta h_{S_{t}E_{a}}}\; & \left({\hat{h}}_{S_{t}E_{a}}+\Delta h_{S_{t}E_{a}}\right)Q_{S_{t}2}{\left({\hat{h}}_{S_{t}E_{a}}+\Delta h_{S_{t}E_{a}}\right)}^{†} \end{array}$$

(24) $$\begin{array}{cc} s.t. & \frac{\mathrm{Tr}(H_{S_{t}S_{r}}Q_{S_{t}2}H_{S_{t}S_{r}}^{†})}{N_{A}}=\gamma_{S_{r}} \end{array}$$

(25) $$\begin{array}{cc} & h_{S_{t}P_{r}}Q_{S_{t}2}h_{S_{t}P_{r}}^{†}=0 \end{array}$$

**Proposition 1.**  *Problem Equations (23)–(25) are equivalent to the following problem, which is given by:*

(26) $$\begin{array}{cc} \min_{Q_{S_{t}2},\lambda_{1},\Theta_{1}}\; & \Theta_{1}+{\hat{h}}_{S_{t}E_{a}}Q_{S_{t}2}{\hat{h}}_{S_{t}E_{a}}^{†}+\lambda_{1}\epsilon_{1}^{2} \end{array}$$

(27) $$\begin{array}{cc} s.t. & \left[\begin{array}{cc} \lambda_{1}I-Q_{S_{t}2} & -Q_{S_{t}2}{\hat{h}}_{S_{t}E_{a}}^{†}\\ -{\hat{h}}_{S_{t}E_{a}}Q_{S_{t}2} & \Theta_{1} \end{array}\right]⪰0 \end{array}$$

(28) $$\begin{array}{cc} & \frac{\mathrm{Tr}(H_{S_{t}S_{r}}Q_{S_{t}2}H_{S_{t}S_{r}}^{†})}{N_{A}}=\gamma_{S_{r}} \end{array}$$

(29) $$\begin{array}{cc} & h_{S_{t}P_{r}}Q_{S_{t}2}h_{S_{t}P_{r}}^{†}=0 \end{array}$$

(30) $$\begin{array}{cc} & Q_{S_{t}2}⪰0,\lambda_{1}⪰0,\Theta_{1}⪰0 \end{array}$$

**Proof.**  We first transform problem Equations (23)–(25) into:

(31) $$\begin{array}{cc} \min_{Q_{S_{t}2}}\;\max_{\Delta h_{S_{t}E_{a}}} & v \end{array}$$

(32) $$\begin{array}{cc} s.t. & \left({\hat{h}}_{S_{t}E_{a}}+\Delta h_{S_{t}E_{a}}\right)Q_{S_{t}2}{\left({\hat{h}}_{S_{t}E_{a}}+\Delta h_{S_{t}E_{a}}\right)}^{†}\leq v \end{array}$$

(33) $$\begin{array}{cc} & \frac{\mathrm{Tr}(H_{S_{t}S_{r}}Q_{S_{t}2}H_{S_{t}S_{r}}^{†})}{N_{A}}=\gamma_{S_{r}} \end{array}$$

(34) $$\begin{array}{cc} & h_{S_{t}P_{r}}Q_{S_{t}2}h_{S_{t}P_{r}}^{†}=0 \end{array}$$

(35) $$\begin{array}{cc} & \forall \Delta h_{S_{t}E_{a}}:∥\Delta h_{S_{t}E_{a}}{∥}^{2}\leq {∥\epsilon_{1}∥}^{2} \end{array}$$

This problem is still intractable to solve since Equation (35) consists of infinite constraints. To tackle this problem, we introduce the S-procedure method to turn these constraints into linear matrix inequalities (LMIs) [46].

**Lemma 1**:  *(S-procedure) Suppose P and Q are n-dimensional symmetric matrices, denoted by $P\in S^{N}$ and $Q\in S^{N}$. Additionally, there exists a vector u satisfying $u^{†}Pu>0$. Then, the implication $u^{†}Pu>0\Rightarrow u^{†}Qu>0$ holds if and only if there exists a λ≥0, such that*:

(36) $$Q-\lambda P\in S_{+}^{N}$$

The constraint Equation (32) can also be expressed as:

(37) $$\begin{array}{cc} -{\hat{h}}_{S_{t}E_{a}}Q_{S_{t}2}{\hat{h}}_{S_{t}E_{a}}^{†}-2ℜ({\hat{h}}_{S_{t}E_{a}}Q_{S_{t}2}\Delta h_{S_{t}E_{a}}^{†})\; & \\ -\Delta h_{S_{t}E_{a}}Q_{S_{t}2}\Delta h_{S_{t}E_{a}}^{†}+v & \geq 0 \end{array}$$

By letting $u=(\Delta h_{S_{t}E_{a}},1)$, $u\in C^{1\times (N_{S_{r}}+1)}$, we reformulate Equation (37) as:

(38) $$u^{†}\left(\left[\begin{array}{cc} 0 & 0\\ 0 & v \end{array}\right]-\left[\begin{array}{cc} Q_{S_{t}2} & Q_{S_{t}2}{\hat{h}}_{S_{t}E_{a}}^{†}\\ {\hat{h}}_{S_{t}E_{a}}Q_{S_{t}2} & {\hat{h}}_{S_{t}E_{a}}Q_{S_{t}2}{\hat{h}}_{S_{t}E_{a}}^{†} \end{array}\right]\right)u\geq 0$$

A similar method can be used to rewrite Equation (35), and it can be given by:

(39) $${\left[\begin{array}{c} \Delta h_{S_{t}E_{a}}\\ 1 \end{array}\right]}^{†}\left[\begin{array}{cc} -I & 0\\ 0 & \epsilon_{1}^{2} \end{array}\right]\left[\begin{array}{c} \Delta h_{S_{t}E_{a}}\\ 1 \end{array}\right]\geq 0$$

According to Lemma 1, let $P=\left[\begin{array}{cc} -I & 0\\ 0 & \epsilon_{1}^{2} \end{array}\right]$ and $Q=\left[\begin{array}{cc} -Q_{S_{t}2} & -Q_{S_{t}2}{\hat{h}}_{S_{t}E_{a}}^{†}\\ -{\hat{h}}_{S_{t}E_{a}}Q_{S_{t}2} & v-{\hat{h}}_{S_{t}E_{a}}Q_{S_{t}2}{\hat{h}}_{S_{t}E_{a}}^{†} \end{array}\right]$; Equations (38) and (39) hold if and only if there exists a $\lambda_{1}\geq 0$, such that:

(40) $$\left[\begin{array}{cc} \lambda_{1}I-Q_{S_{t}2} & -Q_{S_{t}2}{\hat{h}}_{S_{t}E_{a}}^{†}\\ -{\hat{h}}_{S_{t}E_{a}}Q_{S_{t}2} & v-{\hat{h}}_{S_{t}E_{a}}Q_{S_{t}2}{\hat{h}}_{S_{t}E_{a}}^{†}-\lambda_{1}\epsilon_{1}^{2} \end{array}\right]⪰0$$

Letting $\Theta_{1}=v-{\hat{h}}_{S_{t}E_{a}}Q_{S_{t}2}{\hat{h}}_{S_{t}E_{a}}^{†}-\lambda_{1}\epsilon_{1}^{2}$, where $\Theta_{1}\geq 0$, problem Equations (23)–(25) can be rewritten as:

(41) $$\begin{array}{cc} \min_{Q_{S_{t}2},\lambda_{1},\Theta_{1}}\; & \Theta_{1}+\mathrm{Tr}\left({\hat{h}}_{S_{t}E_{a}}Q_{S_{t}2}{\hat{h}}_{S_{t}E_{a}}^{†}\right)+\lambda_{1}\epsilon_{1}^{2} \end{array}$$

(42) $$\begin{array}{cc} s.t. & \left[\begin{array}{cc} \lambda_{1}I-Q_{S_{t}2} & -Q_{S_{t}2}{\hat{h}}_{S_{t}E_{a}}^{†}\\ -{\hat{h}}_{S_{t}E_{a}}Q_{S_{t}2} & \Theta_{1} \end{array}\right]⪰0 \end{array}$$

(43) $$\begin{array}{cc} & \frac{\mathrm{Tr}(H_{S_{t}S_{r}}Q_{S_{t}2}H_{S_{t}S_{r}}^{†})}{N_{A}}=\gamma_{S_{r}} \end{array}$$

(44) $$\begin{array}{cc} & h_{S_{t}P_{r}}Q_{S_{t}2}h_{S_{t}P_{r}}^{†}=0 \end{array}$$

(45) $$\begin{array}{cc} & Q_{S_{t}2}⪰0,\lambda_{1}⪰0,\Theta_{1}⪰0 \end{array}$$

which completes the proof.

Problem Equations (41)–(45) are an SDP problem, which consists of a linear objective function together with some LMI constraints. Thus, one can employ software packages, such as SeDuMi [47], to get the optimal solution of $Q_{S_{t}2}$.

As for the part related to $Q_{S_{t}1}$, we also make a relaxation of Equation (17) by setting:

(46) $$\frac{h_{S_{t}P_{r}}Q_{S_{t}1}h_{S_{t}P_{r}}^{†}}{N_{A}}=\gamma_{P_{r}}$$

Additionally, we have:

(47) $$\begin{array}{cc} \min_{Q_{S_{t}1}}\max_{\Delta h_{S_{t}E_{a}}}\; & \left({\hat{h}}_{S_{t}E_{a}}+\Delta h_{S_{t}E_{a}}\right)Q_{S_{t}1}{\left({\hat{h}}_{S_{t}E_{a}}+\Delta h_{S_{t}E_{a}}\right)}^{†} \end{array}$$

(48) $$\begin{array}{cc} s.t. & \frac{h_{S_{t}P_{r}}Q_{S_{t}1}h_{S_{t}P_{r}}^{†}}{N_{A}}=\gamma_{P_{r}} \end{array}$$

**Proposition 2.**  *Problem Equations (47) and (48) are equivalent to the following problem, which is given by:*

(49) $$\begin{array}{cc} \min_{Q_{S_{t}1},\lambda_{2},\Theta_{2}}\; & \Theta_{2}+\mathrm{Tr}\left({\hat{h}}_{S_{t}E_{a}}Q_{S_{t}1}{\hat{h}}_{S_{t}E_{a}}^{†}\right)+\lambda_{2}\epsilon_{1}^{2} \end{array}$$

(50) $$\begin{array}{cc} s.t. & \left[\begin{array}{cc} \lambda_{2}I-Q_{S_{t}1} & -Q_{S_{t}1}{\hat{h}}_{S_{t}E_{a}}^{†}\\ -{\hat{h}}_{S_{t}E_{a}}Q_{S_{t}1} & \Theta_{2} \end{array}\right]⪰0 \end{array}$$

(51) $$\begin{array}{cc} & \frac{h_{S_{t}P_{r}}Q_{S_{t}1}h_{S_{t}P_{r}}^{†}}{N_{A}}=\gamma_{P_{r}} \end{array}$$

(52) $$\begin{array}{cc} & Q_{S_{t}1}⪰0,\lambda_{2}⪰0,\Theta_{2}⪰0 \end{array}$$

**Proof.**  The proof is similar to the proof for Proposition 1, and the details are omitted here.

#### 4.2.2. The Second Stage

In the second stage, we aim to maximize the denominator of Equation (13) to get the optimal solution of $Q_{H_{e}}$. We can formulate this problem as:

(53) $$\begin{array}{cc} \max_{Q_{H_{e}}}\min_{\Delta h_{H_{e}E_{a}}} & \left({\hat{h}}_{H_{e}E_{a}}+\Delta h_{H_{e}E_{a}}\right)Q_{H_{e}}{\left({\hat{h}}_{H_{e}E_{a}}+\Delta h_{H_{e}E_{a}}\right)}^{†} \end{array}$$

(54) $$\begin{array}{cc} s.t. & h_{H_{e}P_{r}}Q_{H_{e}}h_{H_{e}P_{r}}^{†}=0 \end{array}$$

(55) $$\begin{array}{cc} & \eta P_{P_{t}}{∥h_{P_{t}S_{t}}∥}^{2}\geq \mathrm{Tr}\left(Q_{H_{e}}\right) \end{array}$$

**Proposition 3.**  *Problem Equations (53)–(55) are equivalent to:*

(56) $$\begin{array}{cc} \max_{Q_{H_{e}},\lambda_{3},\Theta_{3}}\; & -\Theta_{3}+\mathrm{Tr}\left({\hat{h}}_{S_{t}E_{a}}Q_{S_{t}2}{\hat{h}}_{S_{t}E_{a}}^{†}\right)-\lambda_{3}\epsilon_{2}^{2} \end{array}$$

(57) $$\begin{array}{cc} s.t. & \left[\begin{array}{cc} \lambda_{3}I+Q_{H_{e}} & Q_{H_{e}}{\hat{h}}_{H_{e}E_{a}}^{†}\\ {\hat{h}}_{H_{e}E_{a}}Q_{H_{e}} & \Theta_{3} \end{array}\right]⪰0 \end{array}$$

(58) $$\begin{array}{cc} & h_{H_{e}P_{r}}Q_{H_{e}}h_{H_{e}P_{r}}^{†}=0 \end{array}$$

(59) $$\begin{array}{cc} & \eta P_{P_{t}}{∥h_{P_{t}S_{t}}∥}^{2}\geq \mathrm{Tr}\left(Q_{H_{e}}\right) \end{array}$$

(60) $$\begin{array}{cc} & Q_{H_{e}}⪰0,\lambda_{3}⪰0,\Theta_{3}⪰0 \end{array}$$

*where $\Theta_{3}=-v+{\hat{h}}_{S_{t}E_{a}}Q_{S_{t}2}{\hat{h}}_{S_{t}E_{a}}^{†}-\lambda_{3}\epsilon_{2}^{2}$.*

**Proof.**  The proof follows a similar procedure as that for Proposition 1 and is omitted.

In summary, the optimal solutions of $Q_{S_{t}1}$, $Q_{S_{t}2}$ and $Q_{H_{e}}$ can be obtained by the aforementioned two stages. Note that $\Delta h_{S_{t}E_{a}}$ and $\Delta h_{H_{e}E_{a}}$ do not directly appear in these optimization problems. The `hidden’ worst-case of $\Delta h_{S_{t}E_{a}}$ and $\Delta h_{H_{e}E_{a}}$ can be obtained by using Lagrange duality, and the derivation process is omitted here. A similar approach can be found in [28].

Furthermore, note that we relax the SINR constraints of the primary traffic and the secondary traffic, as shown in Equations (22) and (46). We have the following proposition.

**Proposition 4.**  The optimum of problem Equations (16)–(21) are achieved when Equations (22) and (46) hold.

**Proof.**  First, for given optimal solutions of $Q_{S_{t}1}$, $Q_{S_{t}2}$ and $Q_{H_{e}}$, let $Q_{S_{t}1}=p_{1}{\bar{Q}}_{S_{t}1}$, $Q_{S_{t}2}=p_{2}{\bar{Q}}_{S_{t}2}$ and $Q_{H_{e}}=p_{3}{\bar{Q}}_{H_{e}}$, where ${\bar{Q}}_{S_{t}1}$, ${\bar{Q}}_{S_{t}2}$ and ${\bar{Q}}_{H_{e}}$ are normalized as $\mathrm{Tr}({\bar{Q}}_{S_{t}1})=1$, $\mathrm{Tr}({\bar{Q}}_{S_{t}2})=1$ and $\mathrm{Tr}({\bar{Q}}_{H_{e}})=1$, respectively. Therefore, problem Equations (16)–(21) with respect to $p_{1}$, $p_{2}$ and $p_{3}$ can be written as:

(61) $$\begin{array}{cc} \min_{p_{1},p_{2},p_{3}}\; & \frac{\left({\hat{h}}_{S_{t}E_{a}}+\Delta h_{S_{t}E_{a}}\right)\left(p_{1}{\bar{Q}}_{S_{t}1}+p_{2}{\bar{Q}}_{S_{t}2}\right){\left({\hat{h}}_{S_{t}E_{a}}+\Delta h_{S_{t}E_{a}}\right)}^{†}}{\left({\hat{h}}_{H_{e}E_{a}}+\Delta h_{H_{e}E_{a}}\right)p_{3}{\bar{Q}}_{H_{e}}{\left({\hat{h}}_{H_{e}E_{a}}+\Delta h_{H_{e}E_{a}}\right)}^{†}+N_{A}} \end{array}$$

It can be easily found that if we enhance the SINR of the primary traffic, $p_{1}$ should be raised, which leads to an undesired increment of Ea’s SINR. Additionally, so is the SINR of the secondary traffic.

### 4.3. An Illustrative Example

**Figure 2:**
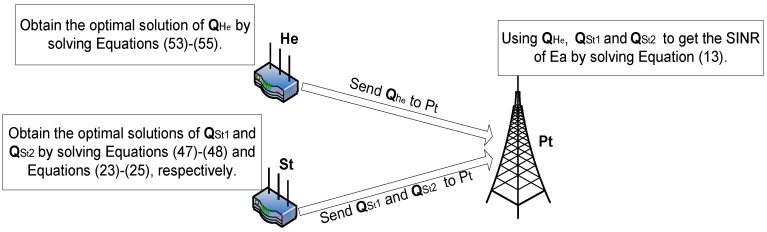
Illustrative example.

To promote better understanding of the proposed jamming scheme, as shown in Figure 2, we take a simple case with a relay St and a helper He. With the purpose of deteriorating the SINR at Ea, St should choose optimal solutions of $Q_{S_{t}1}$ and $Q_{S_{t}2}$. The optimal solution of $Q_{S_{t}2}$ can be obtained by Equations (23)–(25). Similarly, the optimal solution of $Q_{S_{t}1}$ can be obtained by Equations (47) and (48). As for He, it should choose the optimal solution of $Q_{H_{e}}$, and this optimal solution can be obtained by Equations (53)–(55). Using the optimal solutions of $Q_{S_{t}1}$, $Q_{S_{t}2}$ and $Q_{H_{e}}$, the minimum SINR at Ea can be obtained by Equation (13).

## 5. Proposed Auction Framework

In CIoT networks, the primary user and the secondary users will cooperate with each other if their own interests can be met. The primary user wants to choose an optimal helper among multiple secondary users and provides the least amount of energy under a desired degraded SINR at Ea. On the other hand, the helper candidates (secondary users) want to get the most amount of energy by cooperatively jamming Ea. In this paper, we adopt the Vickrey auction [48] to solve the helper selection and corresponding energy allocation problems based on the proposed jamming scheme. In the Vickrey auction, the bidder that submits the lowest bid wins the auction, but pays a price equal to the second-lowest amount bid. Note that if there are more than one lowest bidders, one of them is randomly selected as the winner and pays its own bid (not the second-lowest bid). Unlike some research [36,37,38,39] in this area that assumes to use monetary gains (fictitious currency) to stimulate the participators in an auction, we design a nonmonetary bidding language by a barter-like exchange with the purpose of avoiding the problems associated with money transactions and facilitating the implementation. We choose to adopt the Vickrey auction due to the following two reasons [49]:

- The Vickrey auction guarantees truthfulness. As we know, truthfulness means that for each bidder, reporting true optimal demand is the best strategy regardless of how the other bidders bid. Additionally, it is the critical property for an auction.
- The Vickrey auction is individual rational. Individual rationality means that the utilities of the auctioneer and the winner bidder are always positive. Achieving individual rationality ensures that both the primary user and the secondary users have incentives to participate in the auction.

### 5.1. Mathematical Definitions

In this subsection, the mathematical definitions of the proposed auction framework are presented as follows.

- Auctioneer: primary user Pt.
- Bidder: $Su_{i}\in \mathrm{SU}$, where SU is the set of secondary users with size *N*.
- Characteristic of bidders: A, where $a_{i}=\left(h_{PtSu_{i}},h_{Su_{i}Ea},h_{Su_{i}Pr},H_{Su_{i}Sr}\right)\in A$ denotes the characteristic of bidder $Su_{i}$.
- Valuation of bidder $Su_{i}$: $Su_{i}$’s valuation $v_{i}$ can be formulated as:
  (62) $$v_{i}=E_{i}|\left(\gamma_{E_{a}}=\gamma_{E_{a}}^{exp}\right)$$
  where $\gamma_{E_{a}}$ denotes the SINR at Ea and $\gamma_{E_{a}}^{exp}$ denotes the expected SINR at Ea. $E_{i}$ is the energy that Pt has to provide to $Su_{i}$ to achieve the expected SINR at Ea.
- Bidding of bidders: B, where $b_{i}\in B$ denotes the bidding of bidder $Su_{i}$. $b_{i}$ is related to its individual valuation $v_{i}$.
- Strategy of bidders: Φ, where $\phi_{i}\in \Phi$ describes a strategy of bidder $Su_{i}$.
- Utility of bidder $Su_{i}$: $U_{i}^{Su}\left(\phi_{i},\phi_{-i},a_{i},a_{-i}\right)$, where $\phi_{-i}=\left(\phi_{1},\cdots ,\phi_{i-1},\phi_{i+1},\cdots ,\phi_{N}\right)$ and $a_{-i}=\left(a_{1},\cdots ,a_{i-1},a_{i+1},\cdots ,a_{N}\right)$ denote the vectors of strategies and characteristics of the bidders, except $Su_{i}$, respectively.

### 5.2. Strategy of Bidders

In this subsection, we attempt to investigate the optimal strategies of bidders. For the Vickrey auction, a dominant strategy equilibrium should be achieved to guarantee the system outcome. The definition of the dominant strategy equilibrium is given as follows [49].

**Definition 1.**  The strategy $\Phi^{*}$ achieves the dominant strategy equilibrium, if for every $\phi_{i}^{*}\in \Phi^{*}$, there exists:

(63) $$U_{i}^{Su}\left(\phi_{i}^{*},\phi_{-i},a_{i},a_{-i}\right)\geq U_{i}^{Su}\left(\phi_{i},\phi_{-i},a_{i},a_{-i}\right)$$

As mentioned before, the Vickrey auction guarantees truthfulness, which means that the dominant strategy equilibrium of every bidder is to bid truthfully. Then, we have the following proposition.

**Proposition 5.**  In a Vickrey auction, $\Phi^{*}:\left\{b_{i}=v_{i},i=1,2,\cdots ,N\right\}$ is the only dominant strategy equilibrium.

**Proof.**  Consider $Su_{i}$ bids its true value as $b_{i}=v_{i}$. Here, we assume $b_{i}$ is the winning bid and $b_{k}$ is the second-lowest bid offered by $Su_{k}$. According to the Vickrey auction, Pt pays the second-lowest bid for the winning bidder. Therefore, the utility function of $Su_{i}$ is:

$$U_{i}^{Su}=\left\{\begin{array}{cc} \min\left(b_{0},b_{k}\right)-v_{i}, & if\;\;b_{i}=\min_{l=1,2,\cdots ,N}b_{l}\\ 0, & Otherwise \end{array}\right.$$

$b_{0}$ here denotes the reserve price of Pt. The reserve price means the upper limit of energy that Pt can pay.

(1) $b_{i}\neq v_{i}$, and $Su_{i}$ wins the auction; one can find that the utility of $Su_{i}$ would be unaffected with an unchanged $v_{i}$. Thus, the strategy of making a false bidding cannot improve the bidder’s utility.
(2) $b_{i}\neq v_{i}$, and $Su_{i}$ loses the auction; under this circumstance, the utility of $Su_{i}$ equals zero.

To sum up, $b_{i}=v_{i}$ is the only dominant strategy for bidder *i*, which completes the proof.

For bidder *i*, in order to get its true valuation, it has to calculate the energy $E_{i}$. The required energy of $Su_{i}$ for jamming Ea to achieve $\gamma_{E_{a}}^{exp}$ can be obtained by solving the following problem:

(64) $$\begin{array}{cc} \min_{Q_{H_{e}}}\;\max_{\Delta h_{H_{e}E_{a}}} & \mathrm{Tr}(Q_{H_{e}}) \end{array}$$

(65) $$\begin{array}{cc} s.t.\; & h_{H_{e}P_{r}}Q_{H_{e}}h_{H_{e}P_{r}}^{†}=0 \end{array}$$

(66) $$\begin{array}{cc} & \frac{\left({\hat{h}}_{S_{t}E_{a}}+\Delta h_{S_{t}E_{a}}\right)Q_{S_{t}2}{\left({\hat{h}}_{S_{t}E_{a}}+\Delta h_{S_{t}E_{a}}\right)}^{†}}{\left({\hat{h}}_{H_{e}E_{a}}+\Delta h_{H_{e}E_{a}}\right)Q_{H_{e}}{\left({\hat{h}}_{H_{e}E_{a}}+\Delta h_{H_{e}E_{a}}\right)}^{†}+\left({\hat{h}}_{S_{t}E_{a}}+\Delta h_{S_{t}E_{a}}\right)Q_{S_{t}1}{\left({\hat{h}}_{S_{t}E_{a}}+\Delta h_{S_{t}E_{a}}\right)}^{†}+N_{A}}=\gamma_{E_{a}}^{exp} \end{array}$$

Note that in Equation (66), $\left({\hat{h}}_{S_{t}E_{a}}+\Delta h_{S_{t}E_{a}}\right)Q_{S_{t}2}{\left({\hat{h}}_{S_{t}E_{a}}+\Delta h_{S_{t}E_{a}}\right)}^{†}$ and $\left({\hat{h}}_{S_{t}E_{a}}+\Delta h_{S_{t}E_{a}}\right)Q_{S_{t}1}{\left({\hat{h}}_{S_{t}E_{a}}+\Delta h_{S_{t}E_{a}}\right)}^{†}$ are constants, since $Q_{S_{t}2}$ and $Q_{S_{t}1}$ have been solved in Section 4.2.1. Problem Equations (64)–(66) can be solved using the same approach as problem Equations (53)–(55). Then, the energy that Pt provides to $Su_{i}$, *i.e.*, $E_{i}$ can be formulated as:

(67) $$E_{i}=\frac{\mathrm{Tr}(Q_{H_{e}})}{\eta ∥h_{P_{t}Su_{i}}{∥}^{2}}$$

Besides, one can find that the value of the utility function of the winning bidder is always non-negative. Thus, the individual rationality is always achieved for the secondary users. As for the primary user, the utility function can be written as:

(68) $$U^{Pt}=\gamma_{E_{a}}^{exp}-\gamma_{E_{a}}^{0}$$

where $\gamma_{E_{a}}^{0}$ denotes the SINR at Ea without cooperative jamming. This utility function is always non-negative, which implies the individual rationality of the primary user.

### 5.3. Implementation Details

In this subsection, the implementation of the proposed auction is detailed. Similar to [17,40,41], we assume there exists a common control channel (CCCH) for users to exchange the control information. To be more specific, the transmitter broadcasts common information (some low-rate control or channel state information) via the CCCH. The eavesdropper can also get the control information. However, the message the eavesdropper is interested in is the confidential message sent to the destination. At the beginning of the auction, Pt decides an expected SINR at Ea $\gamma_{E_{a}}^{exp}$ and calculates $Q_{S_{t}1}$ and $Q_{S_{t}2}$ according to Equations (47) and (48) and Equations (23)–(25), respectively. Then, Pt broadcasts an eager-to-help (ETH) frame, which is used to notify the secondary users that the primary user initiates an auction to employ a helper for cooperative jamming. In the ETH frame, $\gamma_{E_{a}}^{exp}$, $Q_{S_{t}1}$ and $Q_{S_{t}2}$ are attached.

Once the ETH frame is received, the secondary users try to participate in the auction by bidding based on their true valuations. Each secondary user calculates its bid $b_{i}$ by using Equations (64)–(67). Then, the secondary user sends an able-to-help (ATH) frame to Pt containing the bidding value (to avoid collision, the frame should be sent after a random backoff value *t*).

Pt waits for a *T* time duration for secondary users’ responses. If no ATH frame is received, Pt considers that no secondary user is available to fulfill its demand. Otherwise, Pt selects the secondary user with the lowest bid as the winning bidder and pays the winning bidder with the second-lowest bid. The lowest bid proposed by the winning bidder indicates that the channel condition between the winner and Pt is good enough. As expected, this will lead to a smaller energy cost at Pt. Note that if only one ATH frame is received, Pt chooses the corresponding bidder as the winning bidder and pays the winning bidder with $b_{0}-b_{i}$. After choosing the winning bidder, Pt broads a ready-to-send (RTS) frame to the secondary users. In the RTS frame, the identity of the winning bidder and the payment results are attached.

## 6. Simulation Study

In this section, we provide some simulation results on the performance of the proposed jamming scheme and the proposed auction framework.

As described in Section 3, Pt, Pr and Ea are all equipped with a single antenna, while the numbers of antennas of St, Sr and He are assumed to be four. We assume that all of the entries in channel coefficient vectors/matrices are independent complex Gaussian random variables with zero mean and unit variance. The power of background noise at all receivers, *i.e.*, St, Sr, Pr and Ea, is assumed to be the same and normalized as $N_{A}=1$, and the transmit power of Pt is defined in dB with respect to the noise power. Unless otherwise specified, the channel uncertainties are assumed to be $∥\Delta h_{S_{t}E_{a}}{∥}^{2}\leq 0.5$ and $∥\Delta h_{H_{e}E_{a}}{∥}^{2}\leq 0.5$; the SINR of the primary traffic and the secondary traffic are set to be $\gamma_{P_{r}}=$ 10 dB and $\gamma_{S_{r}}=$ 10 dB; and the energy harvesting efficiency is given by η=0.1.

### 6.1. Simulation Study of the Proposed Jamming Scheme

We will examine the performance of the proposed jamming scheme under two scenarios: (i) an eavesdropper can only wiretap the primary signal; (ii) a more powerful eavesdropper can wiretap both the primary and the secondary signals. These two scenarios are referred to as Scenario One and Scenario Two, respectively. For the purpose of demonstrating that the proposed jamming scheme can dramatically deteriorate the SINR at Ea, we also examine the scheme without jamming in these two scenarios. We will give particular simulations of the performance of the proposed cooperative jamming scheme under the power constraint of Pt, the channel error bounds and the coefficient of energy harvesting efficiency, respectively.

**Figure 3:**
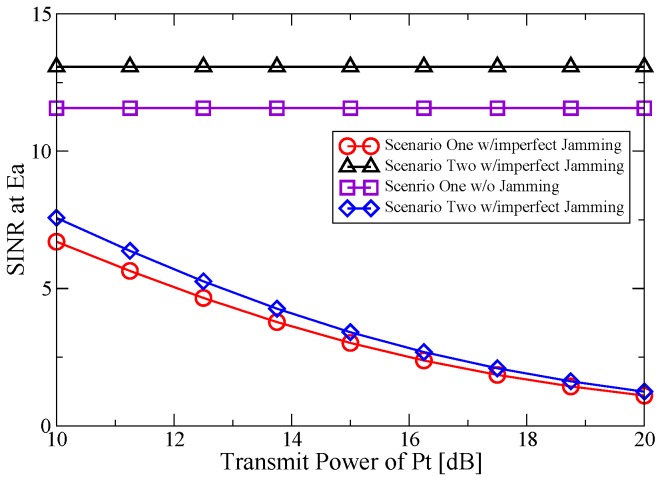
SINR at Ea *vs.* transmit power of Pt.

Figure 3 shows the SINR at Ea as a function of Pt’s transmit power. One can notice that the proposed jamming scheme can definitely deteriorate the SINR at Ea compared to the case with no jamming in Scenario One and Scenario Two. This is due to the fact that the performance of the no jamming scheme is degraded not only by the channel error between St and Ea, but also by that between He and Ea. What is more, it can be observed that when the transmit power of Pt increases, the SINR at Ea tends to be worse. This can be explained as with the increase of Pt’s transmit power, more transmit power can be harvested by He, which can in turn be used to enhance the jamming effect. It is obvious that there is a trade-off between the transmit power and the security capability. With careful planning and execution, a desired SINR at Ea can be obtained using as little transmit power as possible. In addition, the SINR at Ea in Scenario One is worse than Scenario Two. This is reasonable since in Scenario Two, Ea also has the interest to steal transmitted information of the secondary traffic.

**Figure 4:**
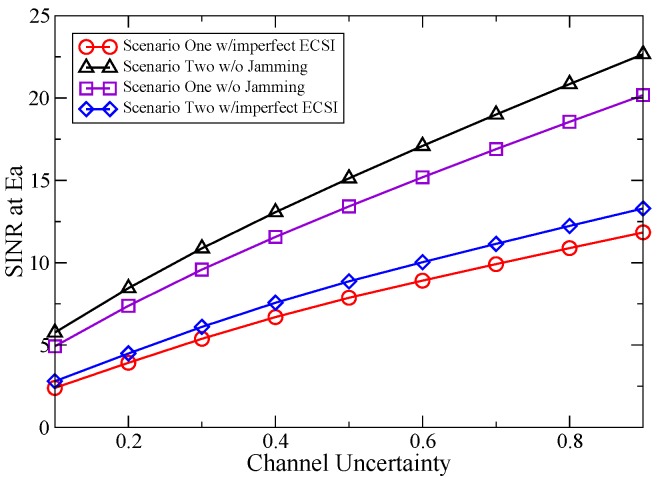
SINR at Ea *vs.* channel uncertainty.

In Figure 4, the SINR at Ea *versus* the channel uncertainty is plotted by assuming $\frac{P_{P_{t}}}{N_{A}}=10$ dB and $\epsilon_{1}^{2}=\epsilon_{2}^{2}$. It can be seen that when the channel uncertainties are zero, the SINR at Ea is very low in the two scenarios. In this case, the proposed robust jamming scheme is not necessary. However, the SINR at Ea will increase with rising $\epsilon_{1}^{2}$ and $\epsilon_{2}^{2}$. This can be understood by the fact that the increase of the channel uncertainties implies worse channel conditions between St and Ea, He and Ea, which leads to a reduction of the jamming performance. The robustness of the proposed jamming scheme is more obvious, and it can enhance the security capability compared to the no jamming case.

**Figure 5:**
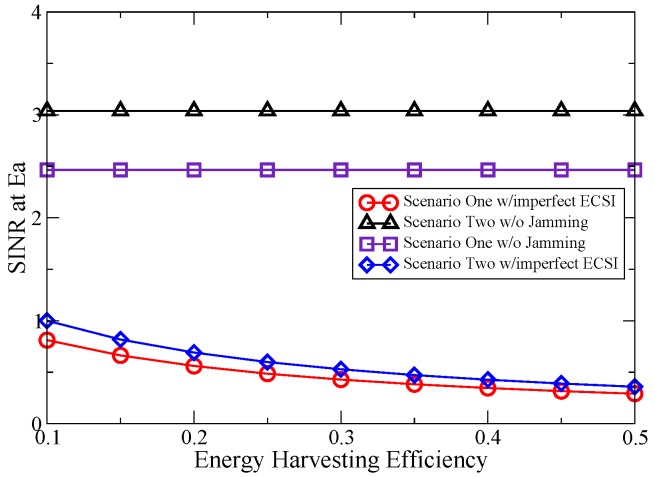
SINR at Ea *vs.* energy harvesting efficiency.

The impact of the energy harvesting efficiency on the SINR at Ea is presented in Figure 5. As shown in Figure 5, the security capability is greatly influenced by the energy harvesting efficiency. With the increase of the energy harvesting efficiency, more transmit power can be seized by He, which leads to a more effective performance. Note that the conversion efficiency is not enough in the experimental measurement [50], resulting in a high power cost at Pt. However, with the improvement of hardware, the conversion efficiency is expected to be increased in the near future. One can also find that the increase of the energy harvesting efficiency leads to a smaller gap between Scenario One and Scenario Two in the proposed jamming scheme. The reason is that a stronger jamming signal reduces the impact of the secondary traffic that is eavesdropped by He.

### 6.2. Simulation Study of the Proposed Auction Framework

**Figure 6:**
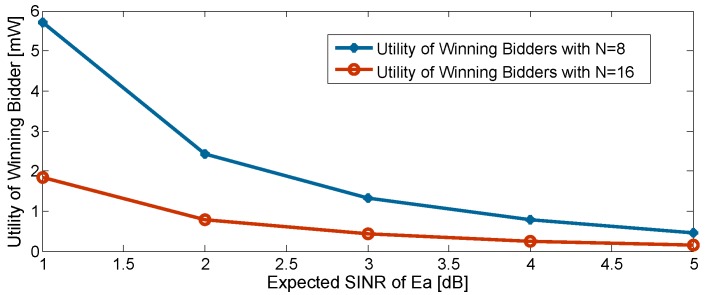
Utility of winning bidder *vs.* expected SINR at Ea.

Figure 6 shows the utility value of the winning bidder as a function of the expected SINR at Ea. It is obvious that the utility value of the winning bidder is positive. This result is expected and confirms the theoretical analysis in Section 5.2. Additionally, a large number of secondary users leads to a smaller utility of the winning bidder. This can be explained as with the increased number of secondary users, the second-lowest bid is more likely to be close to the lowest bid. Furthermore, one can find that a smaller expected SINR at Ea leads to an increase of the utility of the winning bidder. This is because the primary user needs to spend more energy to fulfill its stringent requirement of Ea’s SINR. On the other hand, as can be seen in Figure 6, a higher expected SINR at Ea may cause a no-winner result, regardless of the number of secondary users. In other words, the utility value of the winning bidder tends to be zero. This is because the second-lowest bid is more likely to be close to the lowest bid (winning bid) under a low standard requirement.

**Figure 7:**
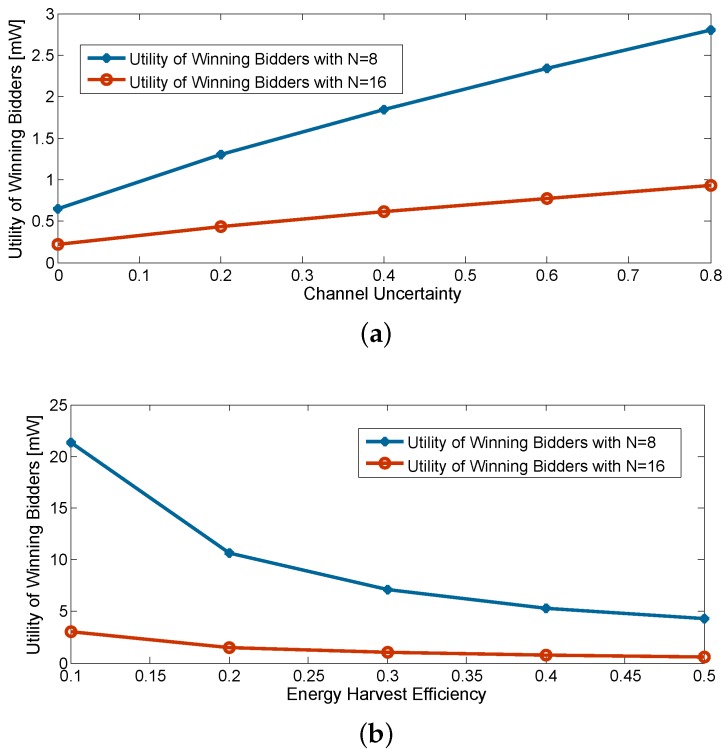
Impacts of channel uncertainty and energy harvest efficiency. (**a**) Utility of winning bidder *vs.* channel uncertainty; (**b**) utility of winning bidder *vs.* energy harvest efficiency.

Figure 7 reports the impacts of the channel uncertainty and the energy harvest efficiency on the utility of winning bidder. The expected SINR at Ea is set to be 0 dB. The utility is observed to be increased under a lower energy harvest efficiency and a bigger channel uncertainty. In the case of low energy harvest efficiency, the secondary users require more energy provided by Pt. With different channel conditions, the lowest bid is more likely to be far from the second-lowest bid. Additionally, so is the large channel uncertainty. Note that the utility of the winning bidder also reduces as the number of the secondary users increases. This can be understood by the fact that as the number of bidders rises, the gap narrows between the second lowest bid and the lowest bid (winning bid).

## 7. Summary and Future Work

This paper addresses the physical layer security issue in CIoT networks by employing cooperative jamming. Firstly, we propose a novel cooperative jamming scheme, in which a secondary user is arbitrarily assigned as the helper to confound the eavesdropper by sending jamming signals. The required energy is harvested from the primary transmitter. The covariance matrix of the helper, as well as the covariance matrices of the relay are optimized to minimize the SINR at the eavesdropper subject to the QoS constraints of the primary traffic and the secondary traffic. Those problems are formulated as a two-stage robust optimization problem and solved by semi-definite programming. Next, we propose to use the Vickrey auction to solve the helper selection and the corresponding energy allocation problems based on the proposed jamming scheme. The goal of the auction framework is to encourage the secondary users to jam the eavesdropper. By investigating the strategy of the secondary user, we prove the truthfulness and individual rationality of the proposed auction framework. We evaluate the performance of the proposed jamming scheme and the auction framework. The evaluation results demonstrate that the SINR at the eavesdropper is remarkably deteriorated. Meanwhile, the helper can be effectively stimulated.

As we know, the IoT device has the characteristics of limited hardware, low-complexity and severe energy constraints. In this paper, the deteriorated SINR at the eavesdropper is achieved at the expense of employing multiple antennas. In future work, we plan to study the trade-off between the security performance and the number of antennas in order to use the smallest number of antennas under a desired security performance. Furthermore, in this paper, the helper node is capable of receiving the confidential information. If the helper is a malicious node, the system security will be severely threatened. The problem of the untrusted helper node should be seriously considered. Finally, it should also be noted that the good performance of the proposed auction framework is achieved at the expense of large time complexity. To reduce the delay time, in future work, we plan to use the optimal stopping theory to design a new helper selection scheme.
